# The impact of childhood sexual abuse on the outcome of intensive trauma-focused treatment for PTSD

**DOI:** 10.1080/20008198.2018.1430962

**Published:** 2018-02-06

**Authors:** Anouk Wagenmans, Agnes Van Minnen, Marieke Sleijpen, Ad De Jongh

**Affiliations:** ^a^ Research Department, PSYTREC, Bilthoven, The Netherlands; ^b^ Radboud University Nijmegen, Behavioural Science Institute (BSI), Nijmegen, The Netherlands; ^c^ Department of Social and Behavioral Sciences, Utrecht University, Utrecht, The Netherlands; ^d^ Department of Social Dentistry and Behavioural Sciences, Academic Centre for Dentistry Amsterdam (ACTA), University of Amsterdam and VU University Amsterdam, Amsterdam, The Netherlands; ^e^ School of Health Sciences, Salford University, Manchester, UK; ^f^ Institute of Health and Society, University of Worcester, UK

**Keywords:** Posttraumatic stress disorder, childhood sexual abuse, intensive trauma-focused treatment, Prolonged Exposure, EMDR therapy, stabilization phase, Trastorno de estrés postraumático, abuso sexual infantil, tratamiento intensivo centrado en el trauma, exposición prolongada, terapia EMDR, fase de estabilización, • No support was found for the hypothesis that a history of (childhood) sexual abuse has a detrimental effect on PTSD treatment outcome.• Patients who had been exposed to a wide variety of traumas, and suffered from multiple comorbidities, benefited from trauma-focused psychotherapy.• Intensive treatment programmes can be effective for patients suffering from severe PTSD in response to a sexual abuse history, regardless of the age at which the traumatic events occurred.

## Abstract

**Background**: It is assumed that PTSD patients with a history of childhood sexual abuse benefit less from trauma-focused treatment than those without such a history.

**Objective**: To test whether the presence of a history of childhood sexual abuse has a negative effect on the outcome of intensive trauma-focused PTSD treatment.

**Method**: PTSD patients, 83% of whom suffered from severe PTSD, took part in a therapy programme consisting of 2 × 4 consecutive days of Prolonged Exposure (PE) and EMDR therapy (eight of each). In between sessions, patients participated in sport activities and psycho-education sessions. No prior stabilization phase was implemented. PTSD symptom scores of clinician-administered and self-administered measures were analysed using the data of 165 consecutive patients. Pre-post differences were compared between four trauma groups; patients with a history of childhood sexual abuse before age 12 (CSA), adolescent sexual abuse (ASA; i.e. sexual abuse between 12 and 18 years of age), sexual abuse (SA) at age 18 and over, or no history of sexual abuse (NSA).

**Results**: Large effect sizes were achieved for PTSD symptom reduction for all trauma groups (Cohen’s *d* = 1.52–2.09). For the Clinical Administered PTSD Scale (CAPS) and the Impact of Event Scale (IES), no differences in treatment outcome were found between the trauma (age) groups. For the PTSD Symptom Scale Self Report (PSS-SR), there were no differences except for one small effect between CSA and NSA.

**Conclusions**: The results do not support the hypothesis that the presence of a history of childhood sexual abuse has a detrimental impact on the outcome of first-line (intensive) trauma-focused treatments for PTSD.

## Abstract (es)

**Planteamiento**: Se asume que los pacientes con TEPT con una historia de abuso sexual infantil se benefician menos del tratamiento centrado en el trauma que aquellos sin dicha historia.

**Objetivo**: Probar si la presencia de una historia de abuso sexual en la infancia tiene un efecto negativo en el resultado del tratamiento intensivo centrado en el trauma para el TEPT.

**Métodos**: Pacientes con TEPT, 83% de los cuales sufrían de TEPT grave, participaron en un programa de terapia que consta de 2 × 4 días consecutivos de exposición prolongada (EP) y terapia EMDR (ocho de cada). Entre sesiones, los pacientes participaron en actividades deportivas y sesiones de psicoeducación. No se implementó una fase de estabilización previa. Las puntuaciones de síntomas de TEPT de las medidas administradas por el clínico y autoadministradas se analizaron usando los datos de 165 pacientes consecutivos. Las diferencias previas se compararon entre cuatro grupos de trauma: pacientes con antecedentes de abuso sexual infantil antes de los 12 años (ASI), abuso sexual adolescente (ASA, es decir, abuso sexual entre 12 y 18 años), abuso sexual (AS) a los 18 años o más, o sin antecedentes de abuso sexual abuso (NAS).

**Resultados**: Se lograron grandes tamaños de efecto en la reducción de síntomas de TEPT para todos los grupos de trauma (*ds* de Cohen = 1,52–2,09). Para la Escala de TEPT Clínica Administrada por el Clínico (CAPS, por sus siglas en inglés) y la Escala de Impacto del Evento (IES, por sus siglas en inglés), no se encontraron diferencias en el resultado del tratamiento entre los grupos de trauma (edad). Para la Escala de autoinforme de síntomas de TEPT (PSS-SR, por sus siglas en inglés), no hubo diferencias, excepto por un pequeño efecto entre ASI y NAS.

**Conclusión**: Los resultados no respaldan la hipótesis de que la presencia de un historial de abuso sexual infantil tenga un impacto perjudicial en el resultado de los tratamientos de primera línea (intensivos) centrados en el trauma para el TEPT.

## 童年性虐待对创伤中心强化治疗PTSD结果的影响

**背景：**经过过童年性虐待的PTSD 病人的治疗效果比没有的病人更差。

**目的：**考察童年性虐待历史是否会对创伤中心强化治疗PTSD结果有负面影响。

**方法：**被试为PTSD 病人，其中80%属于严重PTSD。被试参加一个连续的2X4天的延长暴露（PE）和EMDR 治疗。在治疗之间，病人参加运动活动和心理教育。没有使用前期的稳定阶段。对165个病人的临床医生诊断和自评的PTSD症状分数进行分析。在四个创伤组间比较前后测的差异，四个组包括：在12岁前有童年性虐待经历（CSA）；青少年童年性虐待（ASA，如童年性虐待经历发生在12到18岁）；童年性虐待经历发生在18岁及以上；没有童年性虐待（NSA）。

**结果：**在所有创伤组中 PTSD 症状减轻程度的效应量都较大（Cohen’s *d* = 1.52–2.09）。《临床医师用 PTSD 量表》（CAPS ）和 《事件影响量表》（IES） 测量的治疗结果在四个创伤（年龄）组间没有差异。《自评 PTSD 症状量表》（PSS-SR）的结果也没有差异，除了 CSA 和NSA两组之间的少量区别。

**结果：**结果没有支持原假设，童年性虐待历史并没有对创伤中心的一线（强化）治疗 PTSD 结果造成恶性影响。

It is generally assumed that individuals who have been exposed to cumulative trauma, often of a prolonged and interpersonal nature, are at risk for developing severe forms of posttraumatic stress disorder (PTSD; American Psychiatric Association, 2013; Herman, 1992). Victims of childhood sexual abuse (CSA) are thought to be vulnerable in this regard, because of the interpersonal and repetitive nature of CSA (Cloitre et al., 2012) and because the abuse might disrupt the normal mental development deemed necessary for healthy emotion regulation (Shipman, Zeman, Penza, & Champion, 2000). It has been argued that those with a history of CSA are at risk for developing disturbances involving emotion regulation, interpersonal relationships, and self-concept, which are considered criteria of ‘Complex PTSD’ (Cloitre et al., 2012; Cloitre, Garvert, Brewin, Bryant, & Maercker, 2013), a diagnosis that is currently being considered for inclusion in the 11th revision of the ICD (Maercker et al., 2013).

In general, trauma-focused psychotherapies are considered best practice for treating patients with PTSD according to international guidelines (see Forbes et al., 2010, for a review). To this end, several meta-analyses have shown that Prolonged Exposure (PE) and Eye Movement Desensitization and Reprocessing (EMDR) therapy are effective treatments (e.g. Chen et al., 2014; Cusack et al., 2016). However, due to the more complex symptom profile that patients with a history of CSA are likely to display, it has been argued that conventional trauma-focused therapies are less suitable for this target group (Cloitre et al., 2012, 2010). According to this view, patients would be insufficiently stable to tolerate regular trauma-focused therapy (Cloitre, Koenen, Cohen, & Han, 2002), possibly leading to reduced effectiveness of treatment. Therefore, in 2012 the Complex Trauma Task Force of the International Society of Traumatic Stress Studies (ISTSS) released ‘The Expert Consensus Treatment Guidelines for Complex PTSD in Adults’, which recommended a phase-based treatment approach for patients with ‘Complex PTSD’. In this approach, it is envisioned that implementation of a ‘stabilizing’ phase, consisting of training in emotion regulation and interpersonal skills, is needed before the second phase, that focuses on the processing of traumatic memories (Cloitre et al., 2012).

In contrast to this line of reasoning, evidence has emerged showing that first-line trauma-focused treatments are effective in treating those with a history of CSA and symptoms of Complex PTSD without prior stabilization or training in emotion regulation (Bongaerts, Van Minnen, & De Jongh, 2017; Van Minnen, Harned, Zoellner, & Mills, 2012). For example, in a study among 110 female veterans with PTSD in a residential treatment setting, no differences in treatment response were found using trauma-focused treatment (i.e. Cognitive Processing Therapy) between those with and without a history of CSA (Walter, Buckley, Simpson, & Chard, 2013). This finding was replicated in a sample of 168 female patients with PTSD relating to sexual or physical abuse in either childhood or adulthood who received brief cognitive-behavioural treatment (Resick, Suvak, & Wells, 2014). However, those who reported the presence of childhood abuse showed a significantly poorer response to cognitive processing therapy without cognitive therapy (‘written account only’) compared to those who did not report childhood abuse, albeit the effects were small.

It should be noted that these studies did not consider the age at which the sexual abuse took place as a factor. This could be relevant, as the nature of individuals’ psychological dysfunction might differ as a function of the age at which the abuse was experienced (Cutajar et al., 2010). To this end, studies examining the effects of adverse childhood events on the severity of PTSD indicate that certain types of experiences at specific sensitive periods have stronger impact on and more predictive strength for symptom development than others. While Schalinski et al. (2016) found sexual abuse at age 12 to be the best predictor of the development of PTSD in adulthood, Schoedl et al. (2010) found that individuals who reported CSA before the age of 12 years were more likely to develop depressive symptoms, whereas those abused after 12 years of age were more likely to develop PTSD. It remains unclear, however, whether the age or developmental period at which a child is abused influences the outcome of treatment for PTSD.

As an effort to reduce PTSD symptoms in a substantially shorter time, short and highly intensive treatment programmes have been developed (e.g. Bongaerts et al., 2017; Ehlers et al., 2014; Hendriks et al., 2010). Treatment results of these programmes are promising, as similar treatment outcomes have been found compared to regular trauma-focused therapies, while there are no reports of symptom exacerbation or increased drop-out rates. Thus, short and intensive treatment programmes could be a valuable addition to the existing range of regular trauma-focused therapies. However, research examining the effects of CSA on treatment outcome of these treatment programmes is scarce.

Clearly, more research is needed to assess the impact of CSA histories on treatment response. Given the existing treatment guidelines on complex trauma histories that recommend a phase-based treatment approach as the ‘optimal treatment strategy’ (Cloitre et al., 2012, p. 12), the chief aim of the present study was to test the hypothesis that a history of CSA would have a detrimental effect on the outcome of intensive trauma-focused treatment for those suffering from PTSD. Because of the possible impact of age at which the abuse occurred on treatment response, we examined the differential effects of three age groups in terms of sexual abuse history: CSA (i.e. sexual abuse before age 12), adolescent sexual abuse (ASA, i.e. sexual abuse between 12 and 18 years of age), and sexual abuse occurring at age 18 and above (SA), allowing for a more comprehensive examination of the impact of CSA on treatment response than in previous research. Accordingly, we tested the hypothesis that individuals who had been exposed to CSA would respond worse to trauma-focused treatment for PTSD than individuals who reported having been sexually abused after childhood (either ASA or SA) or individuals who did not report a history of sexual abuse (NSA). Patients were treated using a highly intensive treatment programme consisting of a combination of two first-line trauma-focussed treatments for PTSD, i.e. PE and EMDR therapy, which were applied within a time window of two weeks and without a preceding stabilization phase in terms of emotion regulation skills training. Both therapies are first-line treatments for PTSD according to international treatment guidelines, but the effectiveness of a combination of these treatments has not previously been examined.

## 1. Method

### 1.1. Participants

**Figure 1.**
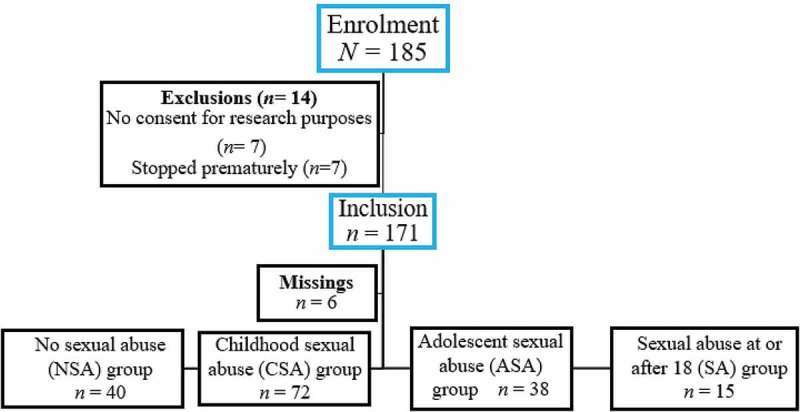
Flowchart of participants.

The study participants were 185 patients (71.9% female; *n* = 133) who were treated between January and August 2016 at the Psychotrauma Expertise Centre (PSYTREC), a mental health centre in Bilthoven, the Netherlands, that provides intensive treatment for patients with PTSD. Of these patients, seven patients did not give informed consent for research purposes due to privacy concerns. Seven patients stopped treatment prematurely (3.8%). Of these 14 participants, all reported exposure to physical abuse, four to sexual abuse, two to work-related trauma, and two to disasters, accidents, and/or war trauma. The 171 remaining participants fulfilled the DSM-IV-TR diagnostic criteria for PTSD (American Psychiatric Association, 2000), but for six patients either the pre- or posttreatment measure was missing. Thus, data for 165 participants, with a mean age at intake of 38.55 years (*SD* = 11.90, range 18–68, 71.5% female), could be used for data analyses. For the self-report measures, complete data were available for respectively *n* = 152 (PSS-SR) and *n *= 127 (IES). See Figure 1 for a flow of participants through the study.

### 1.2. Procedure

After referral by a general practitioner, two intake sessions took place. During the intake sessions, a clinical psychologist assessed whether the patient met the inclusion criteria: (1) a diagnosis of PTSD according to the *DSM–IV–TR* (2000) as established with the CAPS; (2) at least 18 years of age; (3) able to speak and understand the Dutch language sufficiently to undergo treatment; (4) no conviction for sexual assault (to ensure the safety of fellow patients); and (5) no history of a suicide attempt less than three months prior to treatment. Other psychiatric diagnoses (e.g. psychotic disorder or dissociative disorder) were not an exclusion criterion. To establish comorbid psychiatric disorders and suicide risk, patients were assessed using the Mini International Neuropsychiatric Interview (MINI; Lecrubier et al., 1997; Overbeek, Schruers, & Griez, 1999). If the patient met the inclusion criteria, the subject was invited to sign a treatment contract, including informed consent for research purposes. The study was performed in accordance with the precepts and regulations for research as stated in the Declaration of Helsinki and the Dutch Medical Research on Humans Act (WMO) concerning scientific research; that is, all data were collected using the standard assessment instruments and regular monitoring outcome procedure of the PSYTREC mental health centre, the study lacked random allocation, and no additional physical infringement of the physical and/or psychological integrity of the individual was to be expected.

After the intake procedure, the patients received treatment during two consecutive periods of four days per week in the treatment centre. After the first four days of treatment, patients went home for three days, after which they returned for the second four days of treatment. The treatment programme was setup as an outpatient treatment facility, but during the treatment days patients were requested to stay overnight in the centre for practical reasons.

### 1.3. Treatment programme

The patients received two individual treatment sessions of 1.5 hours daily; a PE session of 90 minutes in the morning and an EMDR therapy session of 90 minutes in the afternoon. Treatment was not preceded by a preparation phase. In total, the programme consisted of 16 sessions (24 hours) of trauma-focused therapy. All therapy sessions were provided by psychologists who had received training in EMDR and PE. To verify adherence to the treatment protocols, between the two trauma-focused treatment sessions, all patients were discussed in a team of clinical psychologists and supervisors each day. Every patient was treated by multiple psychologists over the course of their treatment in accordance with the principle of ‘therapist rotation’. Apart from therapy sessions, patients participated in four exercise activities daily, and psycho-education sessions each evening. Further, the patients did not receive any relaxation or emotion regulation skills training prior to the processing of their memories (for the rationale see De Jongh et al., 2016). Only memories of traumatic experiences that fulfilled the DSM-IV Criterion A of PTSD were targeted, starting with the most intrusive memory first.

#### 1.3.1. Prolonged Exposure

The PE sessions followed the slightly modified protocol of Foa’s prolonged exposure protocol (Foa, Hembree, & Rothbaum, 2007). Like in Foa’s protocol, the patient was exposed to the memories of the traumatic events by imagining those as vividly as possible and by describing it aloud in the present tense and in detail for 60 minutes per session. Also, processing of the traumatic memories was included. Because of the intensive treatment format, no homework assignments were given, and therefore the sessions were not recorded. Instead of the in vivo homework assignments, in vivo material, such as pictures, videos, sound fragments, and clothes that reminded the patient of the traumatic event, were incorporated in the PE sessions.

#### 1.3.2. EMDR therapy

During the EMDR therapy sessions, the manualized standard EMDR protocol was used (De Jongh & Ten Broeke, 2013; Shapiro, 2001; for a description, see http://www.emdria.org/?120), Patients were instructed to recall and hold the memory of the traumatic event in mind, while the therapist moved his or her hand in front of the patient’s face to engage the patient in rapid sets of eye movements meant to tax the patient’s working memory. The therapist had the possibility to use additional material, such as a light bar, a clicking sound from left to right, and/or buzzers taken in the hands to maximize the working memory taxation. To address patients’ anticipatory fear and avoidance behaviour, the ‘flashforward protocol’ (Logie & De Jongh, 2014) was applied. During processing, standard cognitive interweaves were applied to open blocked processing (Shapiro, 2001).

#### 1.3.3. Sport programme

In between the therapy sessions, patients participated in an intensive exercise programme, consisting of two forms of exercise in the morning and two in the afternoon. The programme varied from high-intensity to low-intensity indoor and outdoor sports, for example mountain biking, table tennis, and archery. All exercise instructors were specifically trained for this programme and had experience working with PTSD patients.

#### 1.3.4. Psycho-education

In the evenings, psycho-education sessions were provided in groups of 12–14 patients about trauma-related topics, such as avoidance behaviour, re-experiences, negative cognitions and emotions, and how to continue daily life after treatment.

### 1.4. Measures

#### 1.4.1. Trauma exposure

In between the two intake sessions, the patient was asked to complete a modified self-report version of the Interview for Traumatic Events in Childhood (ITEC; Lobbestael, Arntz, Harkema-Schouten, & Bernstein, 2009), a validated measure to assess various dimensions of trauma exposure in multiple contexts. During the second intake session, a psychologist discussed the ITEC form with the patient to assess whether it had been filled in correctly and completely. Based on the information gathered in the intake sessions and from the ITEC, a personal treatment plan was established in collaboration with the patient. For the current study, both ITEC and final treatment plans were analysed to ensure that all cases of report of sexual abuse were included. Patients were categorized into four groups: no history of sexual abuse (NSA), sexual abuse before age 12 (CSA), CSA occurring between 12 and 18 years of age (ASA), and sexual abuse at age 18 and over (SA). The groups were classified based on the age when the sexual assaults took place for the first time. We used the criterion of 12 years because previous studies have shown this to be a critical age in relation to the development of specific psychopathology symptoms (e.g. Schoedl et al., 2010).

#### 1.4.2. Comorbid psychiatric disorders

Patients were assessed for comorbid psychiatric disorders and suicide risk with the Mini International Neuropsychiatric Interview (MINI; Lecrubier et al., 1997; Overbeek et al., 1999). The MINI is a structured, well-validated diagnostic interview that assesses diagnostic criteria of the *DSM-IV-TR* (Van Vliet & De Beurs, 2006).

#### 1.4.3. PTSD symptoms

To assess the change of PTSD symptoms over the course of treatment, the total scores of the Clinician-Administered PTSD Scale pre- and posttreatment were used as a primary outcome measure. In addition, the PTSD Symptom Scale-Self Report pre- and posttreatment and the Impact of Event Scale (each treatment day and the three days in between the first and the second part of the eight treatment days) were used as self-report outcome measures.

The Dutch version of the Clinician-Administered PTSD Scale (CAPS; Blake, Weathers, & Nagy, 1995; Hovens, Luinge, & Van Minnen, 2005) was used to assess 17 PTSD symptoms in the past week according to the diagnostic criteria of the *DSM–IV–TR* (2000). Each symptom was rated on its frequency and intensity on a 5-point Likert scale (ranging from 0 to 4). Overall severity scores were created by summing the frequency and intensity items (range 0–136). A score of ≥ 65 indicated severe symptoms of PTSD, a score between 45 and 65 indicated moderate symptoms of PTSD, and a score ≤ 45 indicated mild or no symptoms of PTSD (Weathers, Keane, & Davidson, 2001). The Dutch version of the CAPS has good internal consistency and validity (Hovens et al., 1994; Weathers et al., 2001), which was replicated in the current study (α = .85).

The Dutch version of the PTSD Symptom Scale-Self Report (PSS-SR; Foa, Riggs, Dancu, & Rothbaum, 1993; Mol et al., 2005) is a 17-item questionnaire that assesses the severity of PTSD symptoms in the past week based on the diagnostic criteria of the *DSM–IV–TR* (2000). It is rated on a 4-point Likert scale from 0 (*not at all)* to 3 (*very much)* and can be used to obtain a total severity score ranging from 0 to 51, with higher scores reflecting higher PTSD severity. The PSS-SR has satisfactory internal consistency and good concurrent validity (Foa et al., 1993). The internal consistency in the present study was satisfactory (α = .83).

The Dutch Impact of Event Scale (IES; Horowitz, Wilner, & Alvarez, 1979; Van Der Ploeg, Mooren, Kleber, Van Der Velden, & Brom, 2004) was used to index the frequency of posttraumatic stress reactions. We used the IES (15-item version) because the authors of the Dutch validation study of the IES-R (revised) recommended the use of the IES rather than the IES-R as the original IES had a better fit compared to the IES-R (Olde, Kleber, Van Der Hart, & Pop, 2006). The IES is a self-report questionnaire that measures trauma-related intrusions and avoidance behaviour. The frequency of each symptom is scored on a 4-point scale, ranging from ‘not at all’ (0), ‘rarely’ (1), ‘sometimes’ (3), to ‘often’ (5). These can be summed to produce a total IES score (range 0–75), with a higher score indicating a greater level of posttraumatic stress phenomena. The IES was modified for the present study to be able to measure how frequently the symptoms occurred during the previous 24 hours rather than the past seven days. Therefore, the IES was administered every morning of the eight treatment days, and during the three days in between when the patients were at home (i.e. 11 days in total). Cronbach’s alpha for intrusion and avoidance items of the current version were acceptable (intrusion α = .76, avoidance α = .69).

### 1.5. Statistical analysis

If missing data did not exceed 10% of the total number of items of a measure, person mean imputation was performed for the CAPS and PSS-SR (Hawthorne & Elliott, 2005). That means that missing values on a certain variable were replaced by the mean score of the available cases. Participants with more than 10% missing items scores per scale were excluded (Cohen, Cohen, West, & Aiken, 2002). For the IES, a more conservative method was used, by carrying the last observation (e.g. the score of the previous day) forward. We allowed a maximum of two out of 11 total scores on the IES to be missing. Independent *t*-tests and chi-square analyses were conducted to compare baseline differences in demographic variables (age and gender) between the trauma groups. For the CAPS, a general linear model (mixed design ANCOVA) was conducted with trauma group (i.e. sexual abuse in childhood, sexual abuse in adolescence, sexual abuse after childhood, no sexual abuse) as the between-subjects factor, and time (pre- versus posttreatment difference score on the CAPS) as the within-subjects factor. Preliminary analyses indicated no violations of assumptions regarding normality, homogeneity of regression slopes, linearity, and homogeneity of variance for the ANCOVA analysis of the CAPS. To adjust statistically for pretreatment differences between groups, we used the total CAPS score administered during the baseline measurement as a covariate. For the PSS-SR analysis, preliminary analyses indicated that the assumption of homogeneity of regression slopes was violated for the ANCOVA analysis. Therefore, the ANCOVA analysis was deemed unsuitable and a factorial repeated measures ANOVA was conducted, with trauma group as the between-subjects factor and time (total pre- and posttreatment scores on the PSS-SR) as the within-subjects factor. Likewise, for the IES analysis, a factorial repeated measures ANOVA was conducted, including the total IES-scores on the eight days of treatment and the three days in between. In case of violation of the assumption of sphericity in the factorial repeated measures ANOVA, a Greenhouse-Geisser correction was applied (Field, 2009). We used Cohen’s *d* to assess the magnitude of effect, and considered 0.2 or less as a small effect, 0.5 as a medium effect, and 0.8 or greater as a large effect (Cohen, 1988). For all statistical analyses, SPSS version 20 was used, and a significance level of α = .05 (two-sided) was adopted.

## 2. Results

### 2.1. Sample characteristics

The sample had a mean CAPS severity score of 93.03 (*SD *= 15.76; range 47–123). Table 1 shows an overview of sample characteristics. As can be seen, almost all patients fulfilled the criteria for severe PTSD (94.5%, *n *= 156), and reported one or more current comorbid psychiatric disorders (83%, *n *= 137; *M *= 1.67, *SD* = 1.27; range: 1–5). A small proportion of the participants (7.9%, *n *= 13) did not fulfil the criteria for any of the comorbid current psychiatric disorders, or had missing data (9.1%, *n *= 15). Suicide risk according to the MINI was present for the majority of the participants (61.8%, *n *= 102).

**Table 1.** Sample characteristics (*N *= 165).

	NSA	CSA	ASA	SA	Total
Mean age (*SD,* range)	43 (11.7, 18–68)	37 (10.8, 20–62)	37 (12.5, 18–63)	40 (14.0, 20–65)	38 (11.9, 18–68)
Sex (% female)	25.0%	87.5%	84.2%	86.7%	71.5%
Mean CAPS score (*SD*)	91.9 (15.5)	95 (15.0)	90.7 (16.2)	92.4 (19.1)	93.0 (15.8)
PTSD severity (CAPS)					
Mild (score ≤ 45)	0%	0%	0%	0%	0%
Moderate (score between 45–65)	5.0%	4.2%	5.3%	13.3%	5.5%
Severe (score ≥ 65)	95.0%	95.8%	94.7%	86.7%	94.5%
Current comorbidity					
None	2.5%	9.7%	13.2%	0%	7.9%
Depression	72.5%	58.3%	60.5%	73.3%	63.6%
Dysthymia	32.5%	34.7%	34.2%	40.0%	34.5%
Hypomania	7.5%	2.8%	0%	0%	3%
Mania	2.5%	0%	0%	0%	0.6%
Panic disorder	12.5%	13.9%	7.9%	13.3%	12.1%
Agoraphobia	15.0%	11.1%	13.2%	6.7%	12.1%
Social phobia	12.5%	19.4%	21.1%	20.0%	18.2%
Obsessive compulsive disorder	0%	11.1%	7.9%	13.3%	7.9%
Alcohol dependency	27.5%	6.9%	18.4%	13.3%	15.2%
Suicidal risk	60.0%	65.3%	57.9%	60.0%	61.8%
Low	45.9%	48.9%	50.0%	66.7%	50.0%
Moderate	33.3%	27.7%	13.6%	22.2%	25.5%
High	20.8%	23.4%	36.4%	11.1%	24.5%
Trauma exposure					
Sexual abuse	0%	100%	100%	100%	75.8%
Physical abuse	42.5%	80.6%	78.9%	100%	72.7%
Work-related trauma	60.0%	9.7%	13.2%	6.7%	22.4%
Disasters, accidents, war trauma	37.5%	16.7%	10.5%	20.0%	20.6%

CAPS = Clinician-Administered PTSD Scale, NSA = no history of sexual abuse, CSA = childhood sexual abuse before age 12, ASA = adolescent sexual abuse (i.e. sexual abuse between 12 and 18 years of age), SA = sexual abuse at age 18 or over. Current comorbidity and suicidal risk was established with the Mini International Neuropsychiatric Interview (MINI).

### 2.2. Trauma exposure

Chi-square analyses showed that women were more likely to report sexual trauma (91.5%), either in childhood or after childhood, than men (36.2%), $\chi^{2}$ (2) = 56.18, *p < *.001, *w *= .58. Participants had often been exposed to multiple types of traumatic experiences (see Table 1; 75.8% sexual abuse, *n *= 125; physical abuse 72.7%, *n *= 120).

### 2.3. Effects of treatment

Regarding the total sample, repeated measures ANOVA’s indicated significant and large treatment effects of time on the CAPS [*F*(1,164) = 301.67, *p *= .001, Cohen’s *d *= 1.70], the PSS-SR [*F*(1,151) = 194.20, *p *= .001, Cohen’s *d *= 1.35], and the IES [*F*(5.78, 727.91) = 75.97, *p *= .001, Cohen’s *d *= 1.80] (see Table 2).

**Table 2.** Descriptive statistics of the ANOVA’s for the total sample and the ANCOVA’s of the four trauma groups.

	*n*	Pretreatment score^a^ *M* (*SD*)	Posttreatment score^b^ *M* (*SD*)	Difference score *M* (*SD*)	Effect size Cohen’s *d*
**Total sample**					
CAPS	165	93.03 (15.76)	47.07 (34.89)	45.96 (33.99)	1.70
PSS-SR	152	36.14 (7.25)	21.09 (14.08)	15.05 (13.32)	1.35
IES	127	57.46 (9.85)	26.63 (22.25)	29.81 (22.46)	1.80
**Trauma group**					
CAPS					
*NSA*	40	91.93 (15.52)	37.65 (33.81)	54.28 (35.82)	2.09
*CSA*	72	95.00 (15.01)	52.49 (36.87)	42.51 (34.23)	1.52
*ASA*	38	90.71 (16.19)	48.89 (32.27)	41.82 (32.14)	1.66
*SA*	15	92.40 (19.10)	41.53 (31.34)	50.87 (30.97)	2.03
PSS-SR					
*NSA*	39	35.64 (6.69)	16.27 (13.53)	19.37 (13.50)	1.84
*CSA*	63	37.20 (6.99)	23.49 (14.44)	13.71 (13.31)	1.22
*ASA*	36	34.05 (7.80)	22.36 (13.56)	11.69 (10.41)	1.07
*SA*	14	38.08 (7.72)	20.43 (13.19)	17.65 (16.93)	1.69
IES					
*NSA*	30	54.24 (12.35)	20.73 (22.05)	33.51 (22.75)	1.91
*CSA*	53	59.58 (8.38)	29.56 (22.22)	30.02 (23.27)	1.80
*ASA*	30	57.45 (9.76)	28.81 (21.57)	28.64 (18.71)	1.74
*SA*	14	56.40 (7.93)	23.50 (23.72)	32.90 (24.15)	1.93

CAPS = Clinician-Administered PTSD Scale, PSS-SR = PTSD Symptom Scale-Self Report,

IES = Impact of Event Scale, NSA = no history of sexual abuse, CSA = childhood sexual abuse before age

12, ASA = adolescent sexual abuse (i.e. sexual abuse between 12 and 18 years of age), SA = sexual abuse

at age 18 or over.

^a^The pretreatment score refers to the CAPS score administered during the baseline measurement, and the

PSS-SR score and IES score administered at the first day of treatment.

^b^The posttreatment score refers to the CAPS and the PSS-SR administered at posttreatment, and the IES

score administered at the last treatment day.

**Figure 2.**
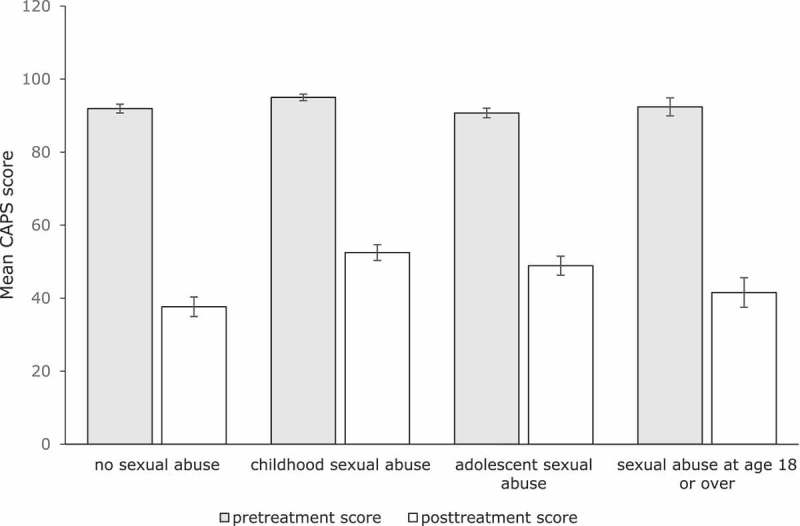
Mean CAPS scores (pre- and posttreatment) for the four trauma groups. Bars indicate one standard error of the mean.

An ANCOVA, with the CAPS pre-treatment score as covariate, indicated that patients with NSA, CSA, ASA, and SA showed equal reductions in CAPS scores over the course of treatment, *F*(3, 160) = 1.53, *p* = .21, $\eta_{p}^{2}$ = .03^1^ (see Figure 2).

**Figure 3.**
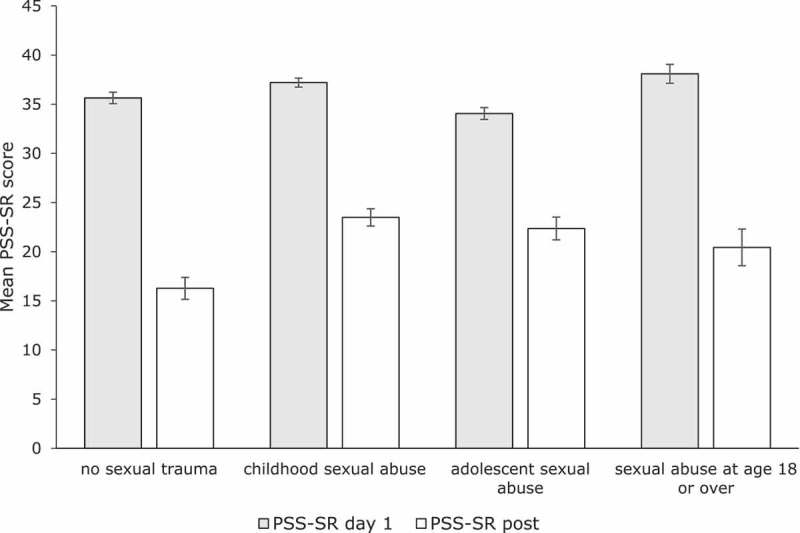
Mean pre- and posttreatment PSS-SR scores for the four trauma groups. Bars indicate one standard error of the mean.

Regarding the PSS-SR, a factorial repeated measures ANOVA that analysed differences between the four trauma groups on PSS-SR total scores of day 1 and posttreatment, showed a main effect of time *F*(1,148) = 161.21, *p *< .001, $\eta_{p}^{2}$ = .52, and a borderline significant interaction effect, *F*(3, 148) = 2.6, *p *= . 05, $\eta_{p}^{2}$ = .05 (see Figure 3). Pairwise comparisons showed that the CSA group (*MD *= 13.72) had lower difference scores on the PSS-SR than the NSA group (*MD *= 19.37), *p *= .02, between-group effect size Cohen’s *d *= 0.43.

**Figure 4.**
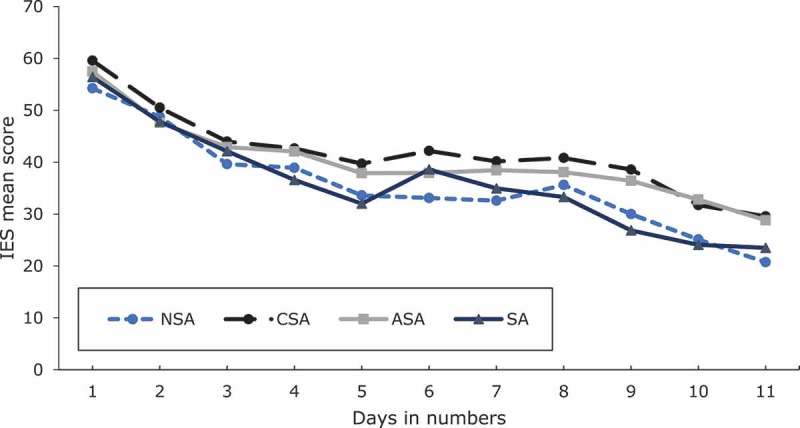
Mean IES scores across the treatment of individuals with no history of sexual abuse (NSA), childhood sexual abuse before age 12 (CSA), adolescent sexual abuse (ASA; i.e. sexual abuse between 12 and 18 years of age), or sexual abuse (SA) at age 18 or over. Days 1–4 and 8–11 refer to treatment days, whereas days 5–7 refer to the days when patients are at home.

A factorial repeated measures ANOVA that investigated differences between the four trauma groups with the 11 IES scores as the dependent variables showed a main effect of time, *F*(5.79, 712.52) = 64.17, *p *< .001, $\eta_{p}^{2}$ = .34, and no time by group interaction, *F*(17.38, 712.52) = 0.70, *p *= .81 (see Figure 4). Table 2 shows an overview of the pre- and posttreatment scores for the four measures per trauma group.

## 3. Discussion

The study reports significant reductions in PTSD symptoms over the course of the treatment using a combination of PE and EMDR therapy within a short and intensive treatment programme on both clinician-administered and self-administered PTSD severity measures. Most importantly, the present study failed to support the hypothesis that a history of sexual abuse has a detrimental effect on treatment outcome. Rather, the results indicate that the patients, of whom the majority reported exposure to a wide variety of traumas and who suffered from multiple comorbidities, benefited strongly from trauma-focused psychotherapy without a stabilization phase. Although our results are at odds with the widely held belief in the trauma field that patients with a history of CSA do worse in trauma-focused treatments than other groups of patients (Cloitre et al., 2012), these are in line with those of previous studies showing that patients suffering from PTSD with and without a history of CSA respond well to trauma-focused treatment (i.e. Resick et al., 2014; Stein, Dickstein, Schuster, Litz, & Resick, 2012).

Our results from clinical interviews carried out by different clinicians confirm those from patients’ daily self-rating of PTSD symptoms. However, differences found on one of the self-report measures, the PSS-SR, and the effect sizes of the trauma-groups suggest that individuals without a history of sexual abuse may have benefitted somewhat more from the treatment programme than those without. It should be noted that these differences were moderately small, explaining only 5% of the variance so that the clinical relevance of this finding may therefore be limited. Furthermore, because the difference was only nominally significant it would not have survived correction for multiple comparisons. Its limited relevance is further underscored by the fact that a large effect size for treatment was found for the total group of patients with a history of sexual abuse before the age of 12, suggesting that individuals with such a trauma history do profit from trauma-focused treatment, but may need more treatment sessions to reach the same outcome.

Although the effects of merging two different trauma-focused, evidence-based therapies for PTSD in an intensive format have not been investigated before, it is conceivable that the immediate focus on trauma and the intensive treatment format places a relatively heavy (emotional) burden on the patient, which in turn could lead to treatment compliance problems and dropout. From this perspective, the drop-out rate in the current study of less than 4% is a remarkable finding. This is even more remarkable as regular treatment programmes for PTSD report drop-out rates of approximately 20% (Imel, Laska, Jakupcak, & Simpson, 2013), given the discussion about the need for a stabilization and emotion regulation phase (De Jongh et al., 2016) and the prevailing notion that a history of CSA renders patients more prone to attrition (Cloitre et al., 2002). However, the reason for the low drop-out rate may also be due to more practical aspects of intensive treatment, since within a short time-frame of two weeks less interfering variables may play a role. Further research pertaining to the mechanisms that protect patients from dropping out from intensive treatment is needed.

There were several limitations to this study. First, given that the intensity of the programme is unique in terms of the frequency with which PE and EMDR were applied, the findings may not generalize to other populations and other treatment programmes, including the more naturalistic clinical settings. Second, although this is the first study to include male victims of sexual abuse, the great majority of participants who had experienced sexual abuse were female (76%). This might have affected the results, as gender differences have been reported in the severity of PTSD symptoms and coping strategies (Ullman & Filipas, 2005). Third, a major limitation of the present study was the lack of randomization and control groups, making it difficult to rule out the possibility that the observed improvements during treatment were an artefact of time. Yet, this seems unlikely given the fact that PTSD symptoms usually do not improve over time alone (Morina, Wicherts, Lobbrecht, & Priebe, 2014). A final limitation is the lack of follow-up. Due to the relatively short existence of the treatment centre, insufficient follow-up data were available for analysis at this time. To this end, more controlled research and long-term data are needed to determine the potential benefits of treatments for those with a history of sexual abuse, and to determine whether a non-phase-based approach, as was used in the present study, leads to different treatment outcomes than sequentially organized (i.e. phase-based) interventions for this target group (Cloitre et al., 2010; De Jongh et al., 2016). Also, it would be important to compare the effects of intensive treatments combining PE and EMDR with the effects of routine treatment that is less frequent and uses only one approach. Further, for future studies it is also important to include outcome measures involving emotion regulation disturbances, difficulties with interpersonal relationships, and self-concept (Maercker et al., 2013). This might help to determine which symptoms, besides PTSD, that are typically associated with sexual abuse or Complex PTSD are relevant for responding to intensive trauma-focused treatment. Strengths of the current study include the use of both clinician-administered and self-administered PTSD measures, and diversity of the sample regarding age and nature of traumatic experiences. It should also be noted that the current patient sample was unique regarding its complexity and symptom severity in that a great majority of the patients met the criteria for severe PTSD and suffered from one or more comorbid psychiatric disorders.

In conclusion, the present results suggest that a short and intensive treatment programme can be effective for patients suffering from severe PTSD in response to a sexual abuse history, regardless of the age at which the traumatic experiences occurred. Further, the findings provide further evidence that a history of (childhood) sexual abuse does not have a detrimental effect on the treatment outcome of these patients. These findings suggest that a trauma-focused treatment can be applied without a preparatory phase, which often lasts several months and therefore might delay or impede recovery (Cloitre et al., 2012; De Jongh et al., 2016). Clearly, more research is needed but, based on the current findings, it does not seem justified to exclude patients with a sexual abuse history from undergoing direct trauma-focused treatment.

## Note

1. The results of analyses controlling for the pre-measurement scores (ANCOVA), and those that did not control for baseline differences (ANOVA) did not differ, whereas the correlation between the difference score and the pretest score was low (CAPS r _τ_ = .12, PSS-SR r _τ_ = .097, IES r _τ_ = .16).
